# Beyond Dietary Identity: Habitual Food Consumption, Lifestyle Characteristics, and Mental Health in Adults Following Plant-Based Diets

**DOI:** 10.3390/nu18182997

**Published:** 2026-09-14

**Authors:** Tamara Kuświk, Konrad Janowski, Agnieszka Białek

**Affiliations:** 1 School of Human Sciences, VIZJA University, Okopowa 59, 01-043 Warsaw, Poland; tkuswik1@wp.pl (T.K.); k.janowski@vizja.pl (K.J.); 2 Collegium Medicum, VIZJA University, Okopowa 59, 01-043 Warsaw, Poland; 3 The Kielanowski Institute of Animal Physiology and Nutrition, Polish Academy of Sciences, Instytucka 3, 05-110 Jabłonna, Poland

**Keywords:** plant-based diet, vegetarian diet, dietary identity, habitual food consumption, diet quality, physical activity, anxiety, depressive symptoms, empathy

## Abstract

Background/Objectives: Evidence linking plant-based diets with mental health is mixed. One reason may be that dietary labels do not always reflect habitual food intake and often coincide with other lifestyle characteristics. Methods: We conducted a cross-sectional secondary analysis of 158 Polish adults: 105 omnivores and 53 participants who identified as following plant-based diets (34 vegetarians and 19 vegans). Habitual intake frequencies for 33 food groups and diet-quality indices were derived from KomPAN. Anxiety and depressive symptoms were assessed with HADS-M, empathy with the EQ-Short/SSIE, and physical activity with IPAQ. Self-identified dietary pattern was the primary exposure; meat/fish-abstention definitions were examined in sensitivity analyses. Results: Compared with omnivores, plant-based participants had lower BMI and nHDI scores, higher DQI values, and, among those with valid IPAQ data, higher physical activity. Nineteen of 33 food groups differed after false-discovery-rate correction. We found no statistically significant association between dietary identity and HADS-A or HADS-D in crude, minimally adjusted, or broader sensitivity models; the confidence intervals, however, were too wide to establish equivalence or rule out an association. Of the 53 self-identified plant-based participants, 36 (67.9%) met the meat/fish-abstention criterion and 17 (32.1%) did not. IPAQ analyses included 120 participants, and inverse-probability-weighted sensitivity analyses produced similar HADS estimates. No individual food-frequency variable was associated with HADS-A or HADS-D after multiplicity correction. In exploratory analyses, more frequent vegetable consumption was positively associated with empathy. Conclusions: Dietary identity corresponded to clear differences in habitual food consumption, diet quality, and selected lifestyle characteristics, but we did not detect a statistically significant association with anxiety or depressive symptoms. The observed mismatch between dietary identity and reported behavior highlights the value of measuring both in studies of plant-based diets and psychological outcomes.

## 1. Introduction

Plant-based diets have attracted growing scientific and public-health interest, but the term itself is used in different ways. It can refer broadly to diets centered on plant foods while still including some animal products, or more narrowly to vegetarian diets that exclude meat and seafood. Vegetarian diets are themselves diverse: vegan diets exclude animal-derived foods, whereas lacto-, ovo-, and lacto-ovo-vegetarian diets include selected dairy products and/or eggs [1,2]. Pesco-, pollo-, flexitarian, and semi-vegetarian patterns retain fish, poultry, or occasional meat and therefore differ from vegetarian diets defined by complete avoidance of meat and seafood [2]. These distinctions matter because a dietary label may not fully describe what a person habitually eats, especially when the research question extends beyond the simple presence or absence of particular animal foods.

The literature on vegetarian and vegan diets and mental health is similarly difficult to interpret. Iguacel et al. [3], in a systematic review and meta-analysis, reported heterogeneous findings for depression, anxiety, and other psychological outcomes and emphasized important limitations in the available evidence. Other observational studies and reviews have reached similarly mixed conclusions [4,5]. Forestell and Nezlek [6], for example, found higher depressive symptoms among vegetarians and semi-vegetarians than among omnivores. Such comparisons cannot show whether diet preceded the psychological symptoms, nor can they separate the role of diet from differences in nutritional adequacy, personality, motivation, health behavior, or other characteristics of the groups being compared. The problem becomes even more pronounced when broad categories such as semi-vegetarian and strictly vegetarian diets are analyzed together. Dietary labels alone may therefore be too coarse to capture the exposures that are relevant to psychological functioning.

One way to address this problem is to look beyond dietary identity and consider the quality and composition of the diet itself [7,8]. In a cross-sectional study of 1949 adults, Millar et al. [9] found that greater adherence to overall and healthful plant-based dietary indices was associated with fewer depressive symptoms, and that overall plant-based diet adherence was inversely associated with anxiety. At the same time, a diet can be strongly plant-oriented without being vegetarian. The Mediterranean diet, for instance, emphasizes vegetables, fruits, legumes, whole grains, nuts, and olive oil but usually includes fish, poultry, dairy products, and limited red meat. Reviews have associated greater adherence to this pattern with potentially favorable mental-health outcomes, although causal evidence remains incomplete [10,11]. This suggests that psychological outcomes may relate more closely to the foods and overall quality of a dietary pattern than to a simple vegetarian-versus-omnivorous label.

Diet also sits within a wider pattern of everyday health behavior [12,13]. Adherence to healthful or unhealthful plant-based dietary patterns may coincide with differences in physical activity, smoking, alcohol consumption, and BMI [9]. In observational studies, these behaviors can make dietary-group comparisons difficult to interpret because the dietary measure may partly reflect a broader lifestyle rather than an isolated exposure. Examining diet alongside physical activity, BMI, and other lifestyle characteristics can therefore provide a more complete description of the groups being compared.

Psychosocial factors may be part of the same picture. People adopt vegetarian diets for different reasons, including health, environmental concerns, and animal welfare [14]. A systematic review comparing vegetarians and omnivores reported differences in several psychological domains, including values, personality-related characteristics, and some aspects of empathy, although the findings varied considerably across studies [15]. We therefore considered empathy alongside anxiety and depressive symptoms, not as a presumed consequence of diet, but as one aspect of the psychosocial context in which dietary choices may occur.

How dietary status is defined is another source of uncertainty. Plant-based, vegetarian, and related categories have been used inconsistently across studies, which limits comparability and can lead to misclassification [1]. Comparing self-identification with reported habitual food consumption can help show whether conclusions depend on the definition used.

We therefore compared Polish adults who identified as omnivorous or plant-based using several complementary measures: dietary identity, habitual frequencies of 33 food groups, composite diet-quality indices, physical activity, BMI, nutrition knowledge, anxiety and depressive symptoms, and empathy. We asked whether plant-based dietary identity corresponded to a distinct food-consumption and lifestyle profile; whether dietary identity or individual food-consumption frequencies were associated with anxiety and depressive symptoms; and whether the findings changed when dietary status was defined by reported meat/fish abstention. We also explored associations between habitual food-consumption frequencies and empathy. This approach allowed self-identification and reported dietary behavior to be examined separately rather than assuming that the two were interchangeable.

## 2. Materials and Methods

### 2.1. Study Design and Participants

This cross-sectional secondary analysis used a previously collected, unpublished dataset from adults living in Poland. Participants were recruited online through internet forums and social-media groups, including communities focused on vegetarian, vegan, and healthy eating. The study invitation linked to an online questionnaire and could be shared further by participants. Respondents received study information and provided electronic informed consent before accessing the questionnaire. No directly identifying personal data were collected.

**Figure 1:**
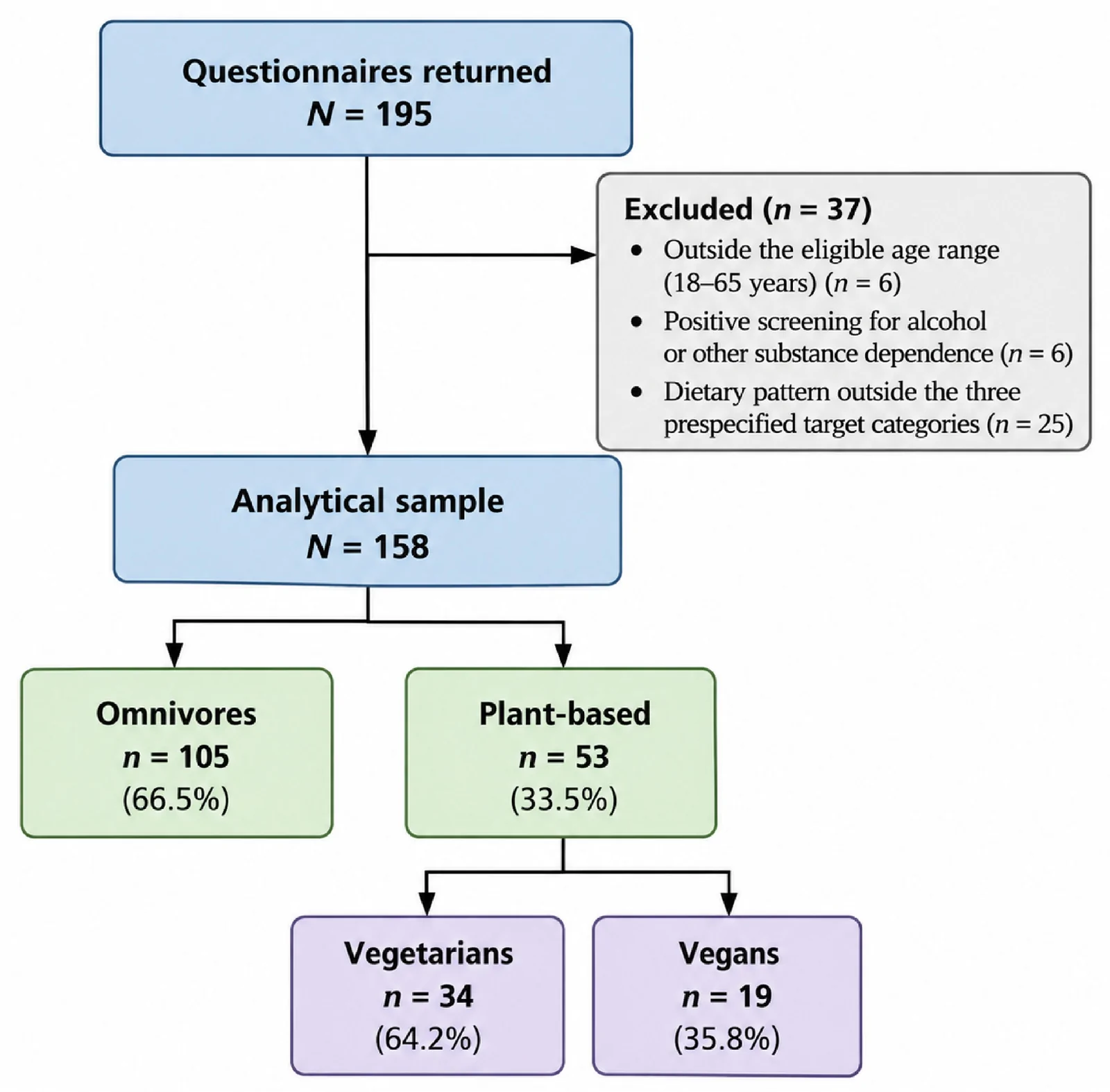
Reconstructed participant flow from the 195 original questionnaire records to the final analytical sample.

The source dataset contained 195 questionnaire records. Record-level reconstruction of participant selection showed that 6 respondents were outside the eligible age range of 18–65 years, 6 screened positive for alcohol or other substance dependence, and 25 reported a dietary pattern outside the three prespecified target categories. These mutually exclusive exclusions left 158 participants for analysis: 105 self-identified omnivores, 34 vegetarians, and 19 vegans. Vegetarians and vegans were combined into the plant-based group (n = 53) for the primary analyses. Participant flow is shown in Figure 1.

Review of the original records also showed that several clinical conditions listed in the study documentation, including depression, diagnosed psychotic disorders, eating disorders, symptoms suggestive of organic central nervous system damage, and coexisting somatic diseases, had not been used to exclude respondents from the analytical sample. Depression was therefore not an applied exclusion criterion in the 158 participants analyzed here, and HADS-D scores were retained across their observed range.

The study protocol was approved by the Ethics Committee for Scientific Research of the University of Economics and Human Sciences in Warsaw, currently VIZJA University (approval no. 02/06/2024, dated 14 June 2024). Informed consent was obtained electronically before participants were granted access to the study questionnaire.

### 2.2. Dietary Classification

The primary exposure variable was self-identified dietary pattern. Participants reporting an omnivorous diet constituted the reference group, whereas participants identifying as vegetarian or vegan were combined into the plant-based group. Thus, the primary classification represented dietary identity rather than retrospective reclassification based on individual food-frequency responses.

Self-identified dietary status and habitual food-consumption frequency were treated as conceptually distinct measures. The food-frequency component of KomPAN referred to habitual consumption during the preceding year. Consequently, reported consumption of meat or fish was not automatically interpreted as a current classification error, because a respondent’s present dietary identity could differ from dietary behavior during part of the recall period. All such records were retained in the primary identity-based analyses.

Two behavior-based definitions were used in sensitivity analyses. Meat/fish abstention was defined as zero reported habitual consumption of processed meat, red meat, white meat, and fish. The second definition, termed identity-meat/fish concordant plant-based status, required both self-identification as vegetarian or vegan and fulfillment of the meat/fish-abstention criterion. Neither definition required abstention from eggs or dairy products; these foods were examined descriptively but were not components of the behavioral classification. Self-identified dietary status remained the primary analytical exposure, whereas the behavior-based definitions were used to assess classification sensitivity.

### 2.3. Measures

#### 2.3.1. Dietary Intake and Diet Quality

Dietary habits and habitual food consumption were assessed using the KomPAN Dietary Habits and Nutrition Beliefs Questionnaire [16]. The instrument includes sections addressing dietary habits, habitual food-consumption frequency, nutrition-related beliefs and knowledge, lifestyle, and sociodemographic characteristics.

Habitual consumption frequency was assessed for 33 food groups. The categorical KomPAN frequency responses were converted to estimated daily frequencies using the numerical coding applied in the KomPAN scoring framework: 0, 0.06, 0.14, 0.5, 1, and 2 times/day. All 33 food-frequency items were complete for all 158 participants.

The pro-Healthy Diet Index (pHDI) and non-Healthy Diet Index (nHDI) were reconstructed from the raw food-frequency responses according to the KomPAN scoring procedure [17]. The pHDI incorporated 10 food-group components and was calculated as pHDI = (100/20) × sum of the daily consumption frequencies of the 10 pHDI components. The nHDI incorporated 14 food-group components and was calculated as nHDI = (100/28) × sum of the daily consumption frequencies of the 14 nHDI components. Both indices were expressed on a scale from 0 to 100. An overall Diet Quality Index (DQI) was calculated as DQI = pHDI − nHDI, with a theoretical range from −100 to 100 and higher values representing a more favorable balance between health-promoting and less favorable dietary characteristics. In addition to these composite indices, all 33 individual food-frequency variables were retained for food-group-level analyses.

#### 2.3.2. Nutrition Knowledge

Nutrition knowledge was assessed using 25 KomPAN questionnaire items. Correct responses were summed to obtain a total score ranging from 0 to 25, with higher scores indicating greater nutrition knowledge. During data verification, the nutrition-knowledge score was independently reconstructed from the underlying questionnaire items and checked against the stored values. Complete scores were available for all 158 participants.

#### 2.3.3. Anxiety and Depressive Symptoms

Anxiety and depressive symptoms were assessed using the Polish modified version of the Hospital Anxiety and Depression Scale (HADS-M [18]), based on the original HADS developed by Zigmond and Snaith [19]. The HADS-M contains the original seven-item anxiety (HADS-A) and seven-item depression (HADS-D) subscales together with two additional items assessing irritability. In the present study, HADS-A and HADS-D were analyzed as continuous outcomes. Each subscale ranges from 0 to 21, with higher scores indicating greater symptom severity.

During the data audit, both HADS-A and HADS-D scores were independently recalculated from the underlying questionnaire items and verified against the stored values. Complete HADS-A and HADS-D data were available for all 158 participants.

#### 2.3.4. Empathy

Empathy was assessed using the Polish adaptation of the 22-item short form of the Empathy Quotient (EQ-Short [20,21]), referred to in Polish as the Skrócona Skala Ilorazu Empatii (SSIE). The instrument measures general empathizing and encompasses affective and cognitive aspects of empathy.

Participants rated each statement using four response categories. Following the scoring approach used in the Polish validation study, responses were coded on a 0–3 scale, with reverse scoring applied to negatively keyed items. Higher total scores indicated greater empathy.

All individual SSIE items and total scores were available for the complete analytical sample. During the data audit, total SSIE scores were independently reconstructed from the item-level responses and verified against the stored scores.

#### 2.3.5. Physical Activity

Physical activity was assessed using the International Physical Activity Questionnaire (IPAQ), covering vigorous-intensity activity, moderate-intensity activity, walking, and sedentary behavior during the preceding seven days. Physical activity was expressed as total metabolic equivalent task minutes per week (MET-min/week).

Because the original spreadsheet contained broken external references in formulas used to derive MET-min/week variables, the stored derived values were not used. IPAQ metrics were independently reconstructed from the underlying questionnaire responses according to the Guidelines for Data Processing and Analysis of the International Physical Activity Questionnaire (IPAQ)—Short and Long Forms [22].

Only activity bouts lasting at least 10 min were included, reported duration was truncated to a maximum of 180 min per activity category, and records with missing or invalid frequency/duration information or total reported activity exceeding 960 min/day were excluded from IPAQ-based analyses. Implausible responses were not manually corrected.

Following quality control, valid total IPAQ MET-min/week values were available for 120 participants, including 80 omnivorous and 40 plant-based participants; 38 records were excluded from IPAQ-dependent analyses.

#### 2.3.6. Anthropometric and Sociodemographic Variables

Body mass index (BMI) was calculated from self-reported body weight and height as weight in kilograms divided by height in meters squared (kg/m^2^). During the revision audit, BMI was recalculated from the source weight and height values for all participants. For one participant whose stored BMI field was blank, complete source weight and height data permitted deterministic reconstruction of BMI; no BMI value was statistically imputed.

Sociodemographic information included sex, education, employment status, and place of residence. Eligibility covered the age range of 18–65 years; however, exact participant-level age was not collected in a form that permitted continuous age adjustment and therefore could not be included as a model covariate. Smoking status was derived from current- and former-smoking responses and categorized as current, former, or never smoking. Alcohol consumption frequency was obtained from the KomPAN habitual-frequency item and retained using the questionnaire frequency categories and corresponding frequency coding, as appropriate for the analysis.

### 2.4. Data Quality Control and Reconstruction of Derived Variables

Before statistical analysis, the original records and analytical dataset underwent a structured record-level and variable-level audit evaluating participant flow, completeness, coding consistency, logical consistency, derived-score calculations, formula integrity, and concordance between self-identified dietary status and habitual food-frequency responses. The revision audit included the complete set of 195 original questionnaire records and independently reconstructed the transition to the final analytical sample of 158 participants.

Complete source data were available for all 33 KomPAN food-frequency variables, nutrition knowledge, HADS-A, HADS-D, and SSIE. Stored pHDI, nHDI, and DQI fields were not available as usable derived variables and were reconstructed from the raw KomPAN food-frequency responses according to the verified scoring procedure. BMI was verified against source weight and height, including deterministic reconstruction of one blank stored BMI value from complete source measurements. IPAQ-derived variables were independently recalculated from the underlying questionnaire responses rather than relying on the original spreadsheet formulas.

Potential discrepancies between self-identified dietary status and reported habitual meat/fish consumption were retained in the primary dataset and addressed through prespecified behavior-based sensitivity definitions rather than manual recoding. Missing or invalid data were handled on an analysis-specific basis. No statistical imputation of outcome or covariate values was performed. IPAQ-dependent analyses included only records satisfying the predefined IPAQ quality-control criteria, with additional selection-bias and inverse-probability-weighted sensitivity analyses conducted as described below.

### 2.5. Statistical Analysis

Continuous variables were summarized using means and standard deviations (SDs) or medians and interquartile ranges (IQRs), as appropriate. Categorical variables were summarized as counts and percentages. All tests were two-sided.

#### 2.5.1. Primary Between-Group Comparisons

Primary descriptive analyses compared participants self-identifying as omnivorous with those self-identifying as vegetarian or vegan. Continuous variables analyzed parametrically, including BMI, pHDI, nHDI, DQI, nutrition knowledge, HADS-A, HADS-D, and SSIE, were compared using Welch’s *t*-test, with Cohen’s d used to quantify between-group effect size. Because total IPAQ MET-min/week was strongly right-skewed, physical activity was compared using the Mann–Whitney U test with rank-biserial correlation (r_rb) as the effect-size measure; this analysis included the 120 participants with valid IPAQ records. Smoking status and alcohol-consumption frequency were additionally summarized by dietary group. Categorical distributions were compared using chi-square or Fisher’s exact tests, as appropriate, and alcohol frequency was compared using the Mann–Whitney U test.

#### 2.5.2. Habitual Food-Consumption Profiles

Habitual consumption frequencies for each of the 33 KomPAN food groups were compared between dietary groups using Mann–Whitney U tests. Rank-biserial correlations were calculated as effect-size estimates. Because the 33 food-group comparisons constituted a family of related tests, *p* values were adjusted using the Benjamini–Hochberg false discovery rate (FDR) procedure. Statistical evidence for a between-group food-frequency difference after multiplicity correction was defined as FDR-adjusted q < 0.05. For food groups with highly zero-inflated distributions, counts of participants reporting any consumption were additionally inspected to support interpretation of rank-based differences.

#### 2.5.3. Food-Consumption Frequencies and Psychological and Behavioral Outcomes

Exploratory correlation analyses examined associations between each of the 33 habitual food-consumption frequencies and five outcomes: HADS-A, HADS-D, SSIE, BMI, and nutrition knowledge. Associations were quantified using Spearman’s rank correlation coefficient (ρ). Benjamini–Hochberg FDR correction was applied separately within each outcome family of 33 food-frequency correlations. Associations with FDR-adjusted q < 0.05 were considered to survive multiplicity correction.

#### 2.5.4. Multivariable Analyses of Anxiety and Depressive Symptoms

Separate ordinary least-squares (OLS) regression models with HC3 heteroscedasticity-robust standard errors were fitted for HADS-A and HADS-D, which were retained as continuous outcomes in their original scale points. Because the study was cross-sectional, the models were interpreted as association models rather than causal-effect estimates.

Self-identified dietary status was the primary exposure. A crude model included dietary status alone. The revised primary adjusted model was minimally adjusted for sex, selected as a baseline characteristic plausibly antecedent to both dietary identity and mental-health symptoms. Exact age could not be included because participant-level age was unavailable beyond the eligibility range. To assess residual sociodemographic confounding, a sensitivity model additionally incorporated education, employment status, and place of residence. A separate lifestyle sensitivity model incorporated smoking status and alcohol-consumption frequency in addition to sex.

BMI, DQI, and SSIE were not treated as conventional baseline confounders because, in a cross-sectional setting, they may characterize the broader dietary, behavioral, or psychosocial phenotype and may potentially lie downstream of dietary behavior. The model used in the submitted manuscript, dietary status, sex, BMI, DQI, and SSIE, was therefore retained as an additionally adjusted phenotype-expanded sensitivity model rather than as the primary confounder-adjusted model. Continuous predictors included in expanded models were standardized before model fitting.

Classification sensitivity was assessed by repeating the crude and minimally adjusted models after replacing self-identified plant-based status with (1) meat/fish abstention and (2) identity-meat/fish concordant plant-based status. A four-category identity-by-behavior regression model was not fitted because only one self-identified omnivorous participant met the meat/fish-abstention criterion, producing an inadequately small cell for stable multivariable estimation; identity-by-behavior concordance was instead summarized descriptively.

#### 2.5.5. IPAQ Missingness and Physical-Activity Sensitivity Analyses

Valid total IPAQ MET-min/week values were available for 120 participants, whereas 38 records failed the predefined IPAQ quality-control criteria. To assess potential selection associated with IPAQ validity, participants with valid and excluded IPAQ records were compared with respect to dietary identity, sex, education, employment, place of residence, BMI, HADS-A, HADS-D, SSIE, DQI, smoking status, and alcohol-consumption frequency using Welch’s *t*-tests, Mann–Whitney U tests, chi-square tests, or Fisher’s exact tests as appropriate, together with effect-size estimates where applicable.

Physical-activity sensitivity models were fitted in the 120 participants with valid IPAQ data using dietary identity, sex, and log1p-transformed standardized total IPAQ MET-min/week. To further examine whether restriction to valid IPAQ records influenced the dietary-status estimates, stabilized inverse-probability-of-observation weights were estimated from a logistic model including dietary identity, sex, higher educational attainment, employment, residence in a city with more than 500,000 inhabitants, smoking status, and alcohol-consumption frequency. Weight distributions and effective sample size were examined to assess stability and positivity, and the physical-activity models were repeated using the stabilized weights with robust standard errors.

#### 2.5.6. Exploratory Multivariable Analysis of Vegetable Consumption and Empathy

Because the food-frequency screening identified a positive association between habitual vegetable consumption and SSIE that remained significant after FDR correction, this association was examined in a targeted exploratory multivariable analysis. OLS regression with HC3 robust standard errors was used, with SSIE score retained in its original scale points as the outcome. Vegetable-consumption frequency was log1p-transformed and standardized. The primary exploratory model adjusted for self-identified dietary status, sex, and BMI. Sensitivity to dietary classification was evaluated by replacing self-identified dietary status with the two behavior-based classifications. An extended sensitivity model, restricted to participants with valid IPAQ data, additionally adjusted for nHDI, nutrition knowledge, and log1p-transformed standardized total IPAQ MET-min/week. Given the exploratory and cross-sectional nature of this analysis, the vegetable–empathy result was interpreted as an association and not as evidence of a causal relationship.

#### 2.5.7. Statistical Significance, Multiplicity, and Interpretation of Non-Significant Estimates

For analyses not subject to multiplicity correction, statistical significance was defined as a two-sided *p* < 0.05. For the 33 between-group food-frequency comparisons and exploratory food-frequency correlation families, inference was based on Benjamini–Hochberg FDR-adjusted q values, with q < 0.05 considered statistically significant. Confidence intervals were reported for regression estimates and used to characterize statistical precision. Non-significant estimates were interpreted as showing that no statistically significant association was detected; they were not interpreted as demonstrating absence, equivalence, or proof of no association. No post hoc observed-power analysis was used to support null findings.

## 3. Results

### 3.1. Participant Characteristics

The final analytical sample comprised 158 adults: 105 self-identified omnivores and 53 self-identified plant-based participants (34 vegetarians and 19 vegans) (Table 1). Record-level reconstruction of the original 195 questionnaires identified 37 mutually exclusive exclusions: 6 respondents outside the eligible age range, 6 screening positive for alcohol or other substance dependence, and 25 reporting dietary patterns outside the three prespecified target categories (Figure 1). Mean BMI was lower in the plant-based group (23.51 ± 3.66 vs. 24.96 ± 4.61 kg/m^2^; Welch *p* = 0.033; Cohen’s d = −0.34). Smoking status did not differ between groups (*p* = 0.885), and habitual alcohol-consumption frequency was also similar (Mann–Whitney *p* = 0.462).

### 3.2. Diet Quality, Physical Activity, and Psychological Outcomes

Diet-quality, lifestyle, and psychological characteristics are summarized in Table 2. Plant-based participants had lower nHDI scores (7.78 ± 5.57 vs. 14.00 ± 8.56; *p* < 0.001; d = −0.81) and higher DQI values (16.78 ± 9.04 vs. 11.96 ± 12.58; *p* = 0.007; d = 0.42). pHDI and nutrition-knowledge scores did not differ significantly. Among the 120 participants with valid IPAQ records, plant-based participants reported higher physical activity [median 2563.5 vs. 1303.5 MET-min/week; *p* = 0.002; rank-biserial r = 0.34]. No statistically significant between-group differences were detected for HADS-A, HADS-D, or SSIE. Of the 158 participants, 120 (75.9%) met IPAQ quality-control criteria and 38 (24.1%) did not. Participants retained and excluded from IPAQ analyses were similar with respect to dietary identity, sex, employment, residence, BMI, HADS-A, HADS-D, SSIE, DQI, and smoking; educational attainment differed (higher education vs. other, *p* = 0.030; Supplementary Table S4).

### 3.3. Habitual Food-Consumption Profiles

**Figure 2:**
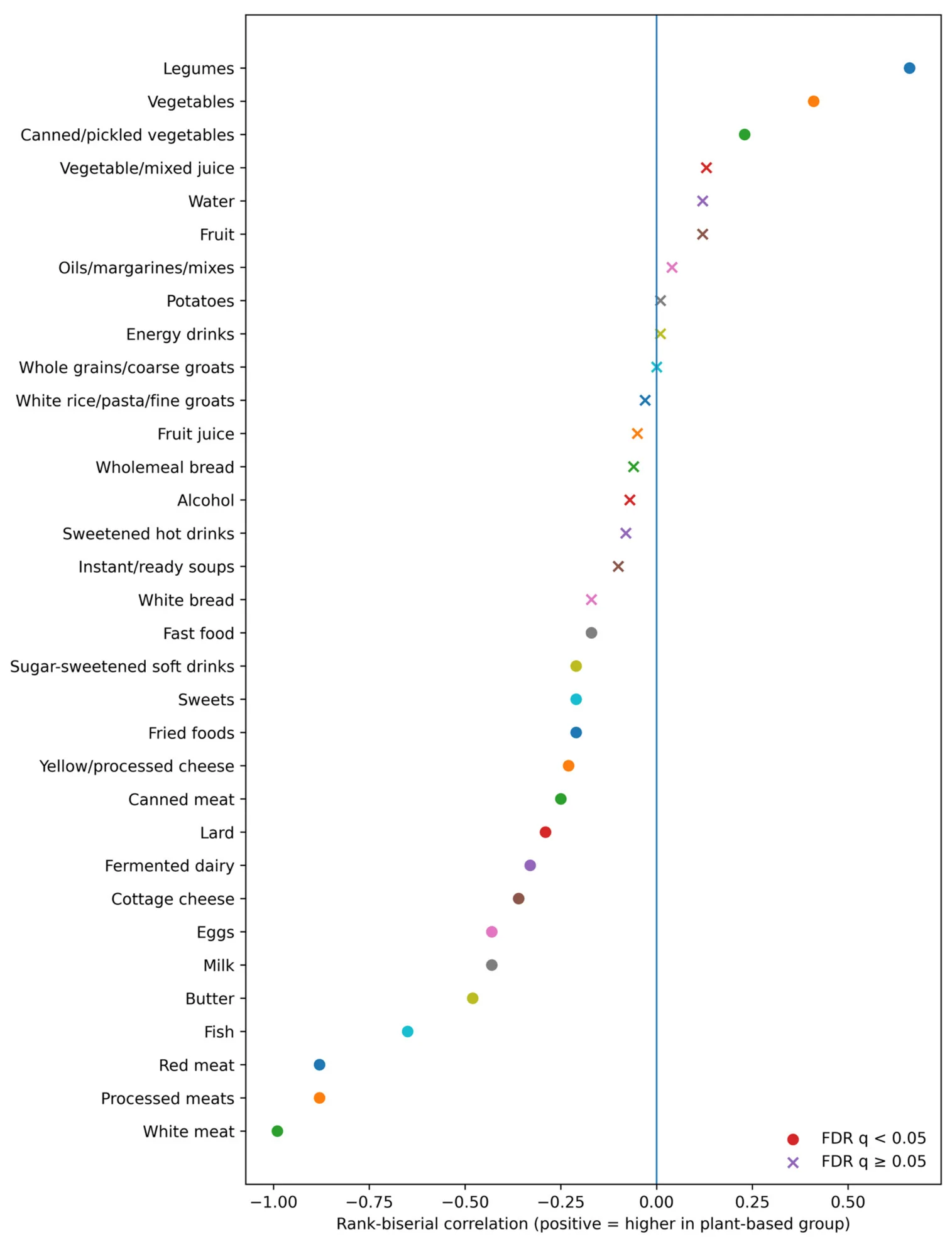
Habitual food-consumption differences between plant-based and omnivorous participants across all 33 KomPAN food-frequency groups. Effect sizes are rank-biserial correlations from Mann–Whitney U tests.

The 33-item KomPAN food-frequency component revealed broad differences in habitual food consumption (Table 3; Supplementary Table S1, Figure 2), with 19 of 33 food groups differing after Benjamini–Hochberg correction. The largest differences included lower reported frequencies of processed meat, red meat, white meat, and fish among plant-based participants, alongside higher legumes and vegetables and lower frequencies of several other foods. For canned meat, both groups had a median of 0.00 [0.00–0.00], but the distributions differed because 26/105 (24.8%) omnivores and 0/53 plant-based participants reported any consumption; the Mann–Whitney comparison therefore remained significant (*p* < 0.001, q < 0.001). Behavioral concordance analyses showed that 36/53 (67.9%) self-identified plant-based participants reported complete abstention from processed meat, red meat, white meat, and fish, whereas 17/53 (32.1%) did not. This criterion was met by 18/34 vegetarians and 18/19 vegans. Detailed identity–behavior cross-tabulations are provided in Supplementary Table S5.

### 3.4. Food-Consumption Frequencies and Psychological Outcomes

Exploratory correlations examined each of the 33 habitual food-consumption frequencies in relation to HADS-A, HADS-D, SSIE, BMI, and nutrition knowledge, with FDR correction within each outcome family. No individual food group was significantly associated with HADS-A or HADS-D after FDR correction. Vegetable-consumption frequency was positively associated with SSIE (Spearman’s ρ = 0.343, unadjusted *p* < 0.001, FDR q < 0.001). Cottage-cheese consumption was positively associated with BMI (ρ = 0.289, FDR q = 0.007). No food-frequency association with nutrition knowledge survived FDR correction.

### 3.5. Multivariable and Classification-Sensitivity Analyses of Mental Health Outcomes

**Figure 3:**
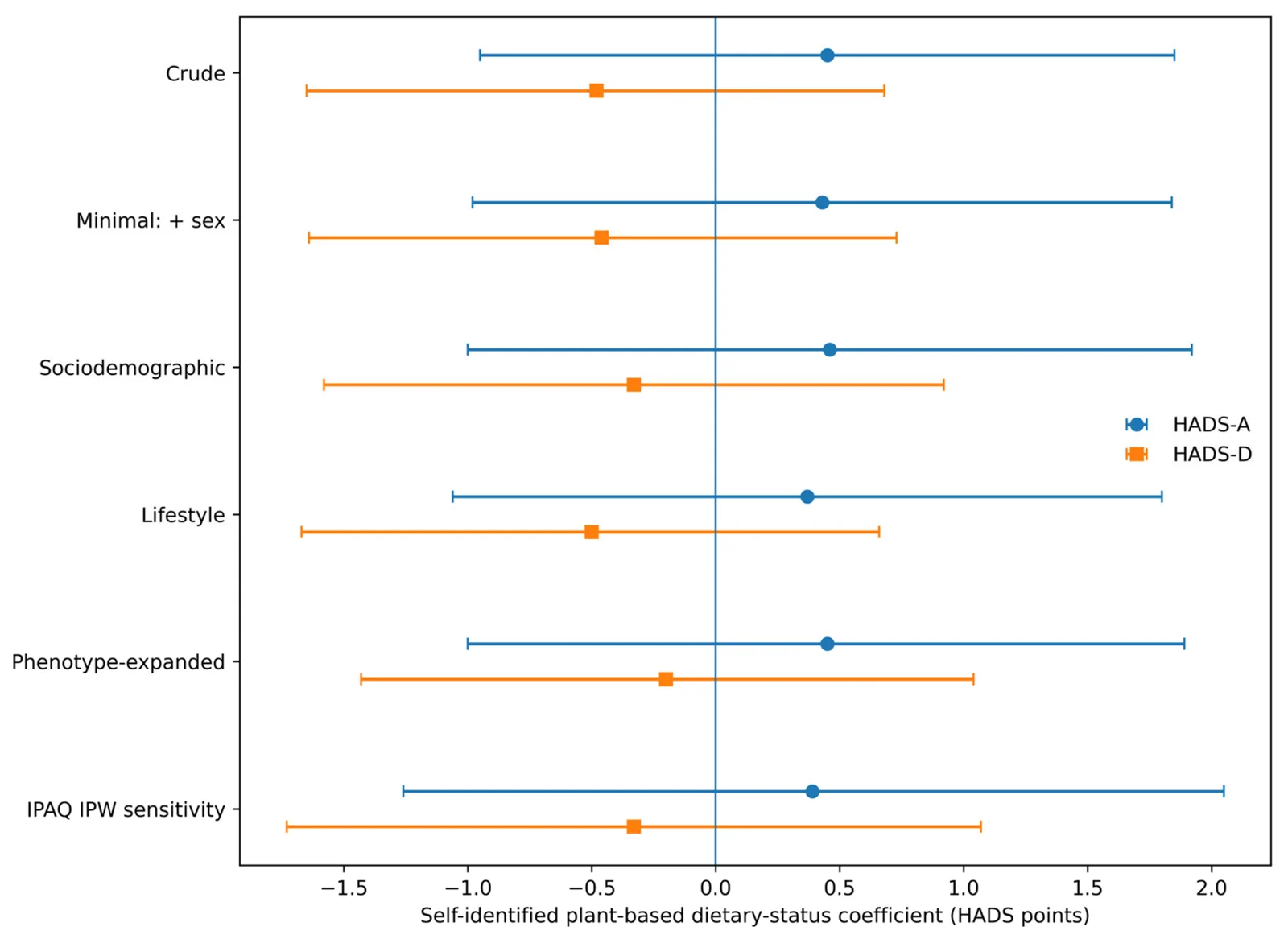
Self-identified dietary-status coefficients for HADS-A and HADS-D across the revised model hierarchy. Points show HC3-robust OLS coefficients and 95% confidence intervals; the vertical line denotes b = 0.

Across the revised hierarchy of HC3-robust OLS models, no statistically significant association was detected between self-identified plant-based dietary status and either HADS-A or HADS-D (Table 4; Supplementary Tables S2 and S6, Figure 3). For HADS-A, the crude coefficient was b = 0.45 (95% CI −0.95 to 1.85; *p* = 0.529) and the minimally adjusted estimate (dietary identity + sex) was b = 0.43 (95% CI −0.98 to 1.84; *p* = 0.549). Estimates were similar in sociodemographic, lifestyle, and phenotype-expanded sensitivity models. For HADS-D, the corresponding crude and minimally adjusted estimates were b = −0.48 (95% CI −1.65 to 0.68; *p* = 0.416) and b = −0.46 (95% CI −1.64 to 0.73; *p* = 0.451), respectively. Alternative meat/fish-abstention classifications likewise did not materially alter inference. In the valid-IPAQ subset, inverse-probability-of-observation weighting produced stable weights (mean 1.006; 99th percentile 1.765; maximum 3.30; effective sample size 112.9) and again yielded no statistically significant dietary-identity estimate for HADS-A [b = 0.39 (95% CI −1.26 to 2.05); *p* = 0.640] or HADS-D [b = −0.33 (95% CI −1.73 to 1.07); *p* = 0.646]. These non-significant estimates were interpreted as lack of detected statistical association, not as evidence of equivalence or proof of absence.

### 3.6. Exploratory Adjusted Association Between Vegetable Consumption and Empathy

**Figure 4:**
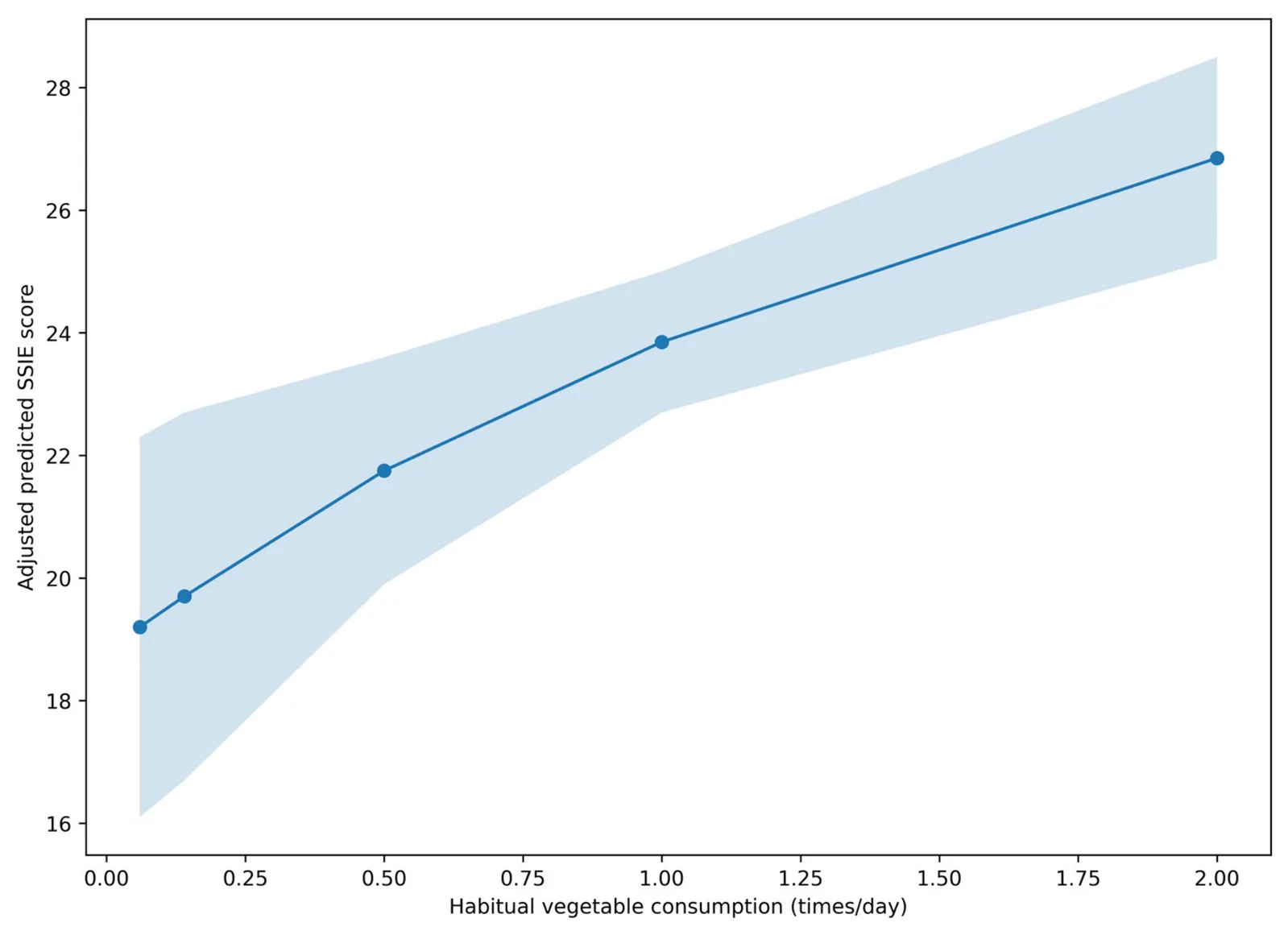
Adjusted marginal association between habitual vegetable consumption and empathy. Predictions are derived from the primary HC3-robust model adjusted for self-identified dietary status, sex, and BMI.

Because vegetable consumption was the only food-frequency correlate of SSIE surviving FDR correction, it was examined in a targeted exploratory model (Table 5, Figure 4). After adjustment for self-identified dietary status, sex, and BMI, higher habitual vegetable consumption was associated with higher SSIE score (b = 2.33 points per 1-SD increase in log-transformed vegetable frequency; 95% CI 1.09 to 3.57; *p* < 0.001). The estimate remained statistically significant in the extended valid-IPAQ sensitivity model (b = 1.76, 95% CI 0.17 to 3.35; *p* = 0.030). Given the exploratory, cross-sectional design, this finding is reported as an association only and should not be interpreted causally.

## 4. Discussion

This study compared dietary behavior, lifestyle, and psychological characteristics in adults who identified as following omnivorous or plant-based diets. The clearest differences were dietary. Plant-based participants differed from omnivores in 19 of 33 food groups after FDR correction, had lower nHDI and higher DQI values, and reported higher physical activity among those with valid IPAQ data. In contrast, we did not detect a statistically significant association between dietary identity and HADS anxiety or depressive symptoms in either the primary or sensitivity analyses. We also found a notable mismatch between dietary identity and reported meat/fish consumption, while the positive association between vegetable consumption and empathy emerged only in exploratory analyses and should be interpreted accordingly.

The mismatch between dietary identity and reported behavior deserves attention. Of the 53 participants who identified as vegetarian or vegan, 36 (67.9%) reported no habitual consumption of processed meat, red meat, white meat, or fish, whereas 17 (32.1%) did not meet this criterion. Most of the discordance occurred among self-identified vegetarians and involved reported fish consumption; 18 of 19 self-identified vegans met the meat/fish-abstention criterion. Because the KomPAN food-frequency questions referred to the preceding year, these responses cannot automatically be treated as errors in current self-identification. A participant may have changed dietary patterns during the recall period. Our behavioral definition also concerned meat and fish only and did not require avoidance of eggs or dairy products. For these reasons, self-identification and reported meat/fish abstention are better viewed as related but distinct descriptions of diet rather than competing definitions of a single ‘true’ dietary status.

The dietary differences extended well beyond meat and fish. Plant-based participants ate legumes and vegetables more frequently and reported less frequent consumption of fast food, fried foods, sweets, and sugar-sweetened beverages. Their lower nHDI and higher DQI values were consistent with this pattern, whereas pHDI did not differ significantly between groups. These results fit with earlier work showing substantial variation both between and within self-defined dietary groups [12,13,23]. They also illustrate why the label ‘plant-based’ cannot be assumed to represent a uniform diet. In our sample, it identified a group with a broader pattern of different food choices, but it still did not fully describe individual habitual intake.

We found no statistically significant association between dietary identity and either HADS-A or HADS-D. This was true in the crude and minimally adjusted models and remained so in the sociodemographic, lifestyle, phenotype-expanded, IPAQ, and inverse-probability-weighted sensitivity analyses. Replacing self-identified status with meat/fish abstention or with identity-meat/fish concordant plant-based status led to the same general interpretation. The wider literature is mixed: studies have reported both less favorable psychological outcomes among people avoiding meat and more favorable outcomes in relation to healthier plant-based dietary patterns [3,4,5,6,7,8,9,24]. Peremin et al. [25], for example, found similar depression, anxiety, and stress scores in vegans and omnivores despite other biological differences between the groups. Nutritional mechanisms, including micronutrient inadequacy, remain possible, but they cannot be inferred from a dietary label. Dietary lithium is a useful example: it is not established as an essential human nutrient, its concentration in foods varies considerably, and grains and vegetables can contribute to dietary exposure [26]. We did not measure nutrient or lithium intake. Our findings therefore do not support a simple adverse or protective interpretation of plant-based dietary identity. They also do not prove that the groups are equivalent: the confidence intervals remain compatible with effects that this sample may have been too small to estimate precisely.

The analyses of individual food groups tell a similar story. Although the two dietary groups differed markedly in what they reported eating, none of the 33 food-consumption frequencies was associated with HADS-A or HADS-D after FDR correction. This makes it less likely that the group comparison simply masked a strong association with one of the measured food categories, but it does not imply that diet is unrelated to mental health. Relevant associations may depend on whole dietary patterns, combinations of nutrients, duration of exposure, or psychosocial context rather than on single food groups [8,9,27]. The same caution applies to the omnivorous category, which can include both diets rich in processed foods and red meat and more plant-forward patterns such as the Mediterranean diet [10,11]. Because KomPAN measures habitual frequency rather than quantities or nutrient intake, and because Mediterranean-diet adherence was not assessed, our data cannot distinguish among these more detailed dietary patterns.

The association between vegetable-consumption frequency and empathy is more tentative. Vegetable consumption was positively related to SSIE in the exploratory screening after FDR correction, and the association remained statistically significant in the targeted adjusted and sensitivity analyses. Overall empathy, however, did not differ significantly between the omnivorous and plant-based groups. Previous studies suggest that food choices may coexist with differences in values, psychosocial characteristics, and some dimensions of empathy [14,15], but our cross-sectional data cannot establish the direction of the association. Personality, ethical concerns, social factors, health consciousness, or other unmeasured characteristics could influence both vegetable consumption and empathy. We therefore regard this result as hypothesis-generating and in need of independent replication.

The study has several strengths. Dietary groups were characterized using more than self-identification alone: the analysis included 33 habitual food-frequency variables, reconstructed diet-quality indices, BMI, physical activity, nutrition knowledge, HADS anxiety and depression scores, and empathy. Participant selection could be reconstructed from all 195 source records, so the final analytical sample of 158 was accounted for explicitly. Rather than recoding participants whose dietary identity and reported intake did not match, we retained those records and quantified the discordance. We also controlled the false discovery rate in the food-frequency analyses and examined the HADS findings across models with different levels of adjustment. Finally, we assessed the possible impact of IPAQ data quality directly. Participants with valid and excluded IPAQ records were similar in dietary identity, psychological outcomes, and most measured characteristics, although educational attainment differed, and inverse-probability-weighted analyses gave a similar interpretation to the observed-case analyses.

Several limitations should also be considered. The cross-sectional design does not establish temporal order or causality, and diet, physical activity, and psychological characteristics were self-reported. KomPAN records habitual food frequency over the preceding year rather than quantitative food, energy, or nutrient intake; this is particularly relevant when current dietary identity does not match reported animal-food consumption during the recall period. Recruitment was based on an online convenience sample and included vegetarian, vegan, and healthy-eating groups. Participants may therefore have been more interested in diet or health than the wider adult population, limiting generalizability. The sample was modest, especially for subgroup and sensitivity analyses, and the confidence intervals around the HADS estimates remain compatible with effects of potentially meaningful size. Exact age was unavailable within the 18–65-year eligibility range, so residual confounding by age cannot be excluded. Other unmeasured socioeconomic, personality, motivational, clinical, and lifestyle factors may also have influenced the results. IPAQ analyses were limited to 120 participants after quality control; education differed between participants retained and excluded, although weighted sensitivity analyses produced similar HADS estimates. The small vegetarian and vegan subgroups also prevented reliable multivariable analysis of every identity-by-behavior combination. Finally, the vegetable-empathy association was exploratory and needs replication, preferably in longitudinal data.

Our results therefore point to a more nuanced view of plant-based eating. In this sample, dietary identity was clearly related to habitual food choices and some lifestyle characteristics, but it was not significantly associated with anxiety or depressive symptoms in the models we examined. The mismatch between self-identification and reported meat/fish consumption also shows why dietary labels should not be used as substitutes for measured intake. Future studies would benefit from larger and more diverse samples, information on the duration and motivation for dietary choices, quantitative dietary assessment, and prospective measurement of behavioral, psychosocial, and clinical factors. These data would allow more precise assessment of whether, and under what circumstances, plant-based dietary patterns are related to mental health.

## 5. Conclusions

In this cross-sectional sample, participants who identified as following plant-based diets had a distinct habitual food-consumption profile, lower nHDI, higher DQI, and, among those with valid IPAQ data, higher physical activity than omnivores. We did not detect a statistically significant association between dietary identity and HADS anxiety or depressive symptoms in crude, minimally adjusted, or sensitivity analyses. The confidence intervals were not sufficiently narrow to interpret these findings as evidence of equivalence or proof that no association exists.

Dietary identity and reported behavior were not fully aligned: 36 of 53 self-identified plant-based participants met the meat/fish-abstention criterion and 17 did not. This reinforces the value of measuring habitual intake rather than relying on dietary labels alone. None of the 33 individual food-consumption frequencies was associated with anxiety or depressive symptoms after multiplicity correction. The positive association between vegetable-consumption frequency and empathy was exploratory and should be treated as hypothesis-generating because its direction and possible residual confounding cannot be resolved in this cross-sectional study.

Plant-based diets are therefore better studied as heterogeneous dietary and lifestyle patterns than as a single binary exposure. Larger and more diverse longitudinal studies that combine dietary identity with quantitative intake, duration and motivation for dietary choices, and detailed lifestyle and psychosocial measures should provide a clearer picture of how these patterns relate to psychological functioning.

## Figures and Tables

**Table 1:** Participant characteristics according to dietary identity.

Variable	Plant-Based (n = 53)	Omnivorous (n = 105)	*p* Value/Effect
Sex: female	46 (86.8%)	87 (82.9%)	0.065
Education: higher	39 (73.6%)	65 (61.9%)	0.393
Employment: permanent/fixed-term	29 (54.7%)	58 (55.2%)	0.050
BMI, kg/m^2^	23.51 ± 3.66	24.96 ± 4.61	0.033; d = −0.34
Smoking: current	14 (26.4%)	24 (22.9%)	
Smoking: former	15 (28.3%)	31 (29.5%)	
Smoking: never	24 (45.3%)	50 (47.6%)	0.885
Alcohol frequency, times/day	0.06 [0.00, 0.06]	0.06 [0.00, 0.06]	0.462

**Table 2:** Diet quality, nutritional knowledge, physical activity, and psychological outcomes.

Variable	Omnivorous	Plant-Based	*p* Value	Effect Size
pHDI (0–100)	25.96 ± 11.34	24.56 ± 7.08	0.344	d = −0.14
nHDI (0–100)	14.00 ± 8.56	7.78 ± 5.57	<0.001	d = −0.81
DQI (−100 to 100)	11.96 ± 12.58	16.78 ± 9.04	0.007	d = 0.42
Nutrition knowledge (0–25)	13.97 ± 4.34	13.25 ± 3.27	0.242	d = −0.18
HADS-A	8.34 ± 3.63	8.79 ± 4.47	0.527	d = 0.11
HADS-D	5.22 ± 3.48	4.74 ± 3.50	0.414	d = −0.14
SSIE	24.41 ± 7.04	25.21 ± 8.47	0.556	d = 0.11
IPAQ total MET-min/week (valid n = 80/40)	1303.50 [720.00, 3096.75]	2563.50 [1668.75, 4522.50]	0.002	r_rb = 0.34

**Table 3:** Habitual food-consumption groups differing after FDR correction.

Food Group	Omnivorous Median [IQR], Times/Day	Plant-Based Median [IQR], Times/Day	Rank-Biserial r	*p* Value	FDR q
Fast food	0.06 [0.06, 0.06]	0.06 [0.06, 0.06]	−0.17	0.027	0.048
Fried foods	0.14 [0.06, 0.50]	0.14 [0.06, 0.50]	−0.21	0.023	0.045
Butter	0.50 [0.06, 1.00]	0.06 [0.00, 0.14]	−0.48	<0.001	<0.001
Lard	0.00 [0.00, 0.06]	0.00 [0.00, 0.00]	−0.29	<0.001	<0.001
Milk	0.50 [0.14, 1.00]	0.06 [0.00, 0.50]	−0.43	<0.001	<0.001
Fermented dairy	0.50 [0.14, 0.50]	0.14 [0.00, 0.50]	−0.33	<0.001	0.001
Cottage cheese	0.14 [0.06, 0.50]	0.06 [0.00, 0.14]	−0.36	<0.001	<0.001
Processed cheese	0.14 [0.06, 0.50]	0.14 [0.00, 0.50]	−0.23	0.017	0.038
Processed meats	0.50 [0.06, 0.50]	0.00 [0.00, 0.00]	−0.88	<0.001	<0.001
Red meat	0.14 [0.06, 0.50]	0.00 [0.00, 0.00]	−0.88	<0.001	<0.001
White meat	0.50 [0.14, 0.50]	0.00 [0.00, 0.00]	−0.99	<0.001	<0.001
Fish	0.06 [0.06, 0.14]	0.00 [0.00, 0.06]	−0.65	<0.001	<0.001
Eggs	0.50 [0.14, 0.50]	0.06 [0.00, 0.50]	−0.43	<0.001	<0.001
Legumes	0.06 [0.06, 0.14]	0.50 [0.14, 0.50]	0.66	<0.001	<0.001
Vegetables	1.00 [0.50, 2.00]	2.00 [1.00, 2.00]	0.41	<0.001	<0.001
Sweets	0.50 [0.14, 0.50]	0.14 [0.06, 0.50]	−0.21	0.025	0.046
Canned meat	0.00 [0.00, 0.00]	0.00 [0.00, 0.00]	−0.25	<0.001	<0.001
Canned/pickled vegetables	0.06 [0.06, 0.14]	0.14 [0.06, 0.50]	0.23	0.013	0.031
Sugar-sweetened soft drinks	0.06 [0.00, 0.06]	0.00 [0.00, 0.06]	−0.21	0.023	0.045

**Table 4:** Robust multivariable sensitivity models for HADS-A and HADS-D.

Outcome	Model	N	b	95% CI	*p* Value
HADS-A	Crude	158	0.45	−0.95 to 1.85	0.529
HADS-A	Minimal: +sex	158	0.43	−0.98 to 1.84	0.549
HADS-A	Sociodemographic	158	0.46	−1.00 to 1.92	0.537
HADS-A	Lifestyle	158	0.37	−1.06 to 1.80	0.616
HADS-A	Phenotype-expanded	158	0.45	−1.00 to 1.89	0.545
HADS-A	IPAQ IPW sensitivity	120	0.39	−1.26 to 2.05	0.640
HADS-D	Crude	158	−0.48	−1.65 to 0.68	0.416
HADS-D	Minimal: +sex	158	−0.46	−1.64 to 0.73	0.451
HADS-D	Sociodemographic	158	−0.33	−1.58 to 0.92	0.602
HADS-D	Lifestyle	158	−0.50	−1.67 to 0.66	0.395
HADS-D	Phenotype-expanded	158	−0.20	−1.43 to 1.04	0.755
HADS-D	IPAQ IPW sensitivity	120	−0.33	−1.73 to 1.07	0.646

**Table 5:** Adjusted association between habitual vegetable consumption and empathy.

Model	Diet Classification Adjustment	N	Vegetable-Frequency b	95% CI Low	95% CI High	*p* Value	R^2^	Adjusted R^2^
Primary adjusted	Self-identified plant-based	158	2.331	1.094	3.568	<0.001	0.128	0.106
Primary adjusted	Meat/fish abstention	158	2.274	1.072	3.477	<0.001	0.125	0.102
Primary adjusted	Identity-meat/fish concordant plant-based status	158	2.232	1.038	3.426	<0.001	0.124	0.101
Extended sensitivity	Self-identified plant-based	120	1.757	0.166	3.348	0.030	0.134	0.080

## Data Availability

The data supporting the findings of this study are available from the corresponding author upon reasonable request.

## Supplementary Materials

The following supporting information can be downloaded at https://www.mdpi.com/article/10.3390/nu18182997/s1: Table S1: All 33 habitual food-consumption comparisons; Table S2: Revised hierarchy of HADS models for self-identified dietary status; Table S3: Sensitivity of HADS estimates to alternative dietary classifications; Table S4: Comparison of participants with valid and excluded IPAQ records; Table S5: Dietary identity and reported animal-food consumption used to assess identity-behavior concordance; Table S6: IPAQ observed-case and inverse-probability-weighted HADS sensitivity analyses; Table S6B: Diagnostics for stabilized inverse-probability-of-observation weights.

## Abbreviations

BMI	Body mass index
DQI	Diet Quality Index
FDR	False discovery rate
HADS-A	Hospital Anxiety and Depression Scale-Anxiety
HADS-D	Hospital Anxiety and Depression Scale-Depression
HADS-M	Hospital Anxiety and Depression Scale-Modified Version
IPAQ	International Physical Activity Questionnaire
MET	Metabolic equivalent task
nHDI	non-Healthy Diet Index
pHDI	pro-Healthy Diet Index
SSIE	Skrócona Skala Ilorazu Empatii/Short Empathy Quotient Scale

## References

[1] Hargreaves S.M., Rosenfeld D.L., Moreira A.V.B., Zandonadi R.P. (2023). Plant-based and vegetarian diets: An overview and definition of these dietary patterns. Eur. J. Nutr..

[2] Gajski G., Gerić M., Jakaša I., Peremin I., Domijan A.-M., Vučić Lovrenčić M., Kežić S., Bituh M., Moraes de Andrade V. (2023). Inflammatory, oxidative and DNA damage status in vegetarians: Is the future of human diet green?. Crit. Rev. Food Sci. Nutr..

[3] Iguacel I., Huybrechts I., Moreno L.A., Michels N. (2021). Vegetarianism and veganism compared with mental health and cognitive outcomes: A systematic review and meta-analysis. Nutr. Rev..

[4] Fazelian S., Sadeghi E., Firouzi S., Haghighatdoost F. (2022). Adherence to the vegetarian diet may increase the risk of depression: A systematic review and meta-analysis of observational studies. Nutr. Rev..

[5] Luque-Martínez A., Ávila-Jiménez Á.F., Reinoso-Espín Á., Araújo-Jiménez M.Á., Martos-Salcedo C.R., González-Domenech P., Jiménez-Fernández S., Martínez-Ruiz V., Cano-Ibáñez N., Rivera-Izquierdo M. (2025). Meat consumption and depression: An updated systematic review and meta-analysis. Nutrients.

[6] Forestell C.A., Nezlek J.B. (2018). Vegetarianism, depression, and the five factor model of personality. Ecol. Food Nutr..

[7] Walsh H., Lee M., Best T. (2023). The association between vegan, vegetarian, and omnivore diet quality and depressive symptoms in adults: A cross-sectional study. Int. J. Environ. Res. Public Health.

[8] Bizzozero-Peroni B., Díaz-Goñi V., Fernández-Rodríguez R., Martínez-Vizcaíno V., Jiménez-López E., Visier-Alfonso M.E., Garrido-Miguel M., Mesas A.E. (2025). Plant-based diets and mental and neurocognitive health outcomes: A systematic review with meta-analysis. Nutr. Rev..

[9] Millar S.R., Perry I.J., Phillips C.M. (2026). Plant-based dietary indices and mental health: A cross-sectional study of a middle- to older-aged population. Eur. J. Nutr..

[10] Ventriglio A., Sancassiani F., Contu M.P., Latorre M., Di Salvatore M., Fornaro M., Bhugra D. (2020). Mediterranean Diet and its Benefits on Health and Mental Health: A Literature Review. Clin. Pract. Epidemiol. Ment. Health.

[11] Akerele C.A., Koralnik L.R., Lafont E., Gilman C., Walsh-Messinger J., Malaspina D. (2025). Nutrition and brain health: Implications of Mediterranean diet elements for psychiatric disorders. Schizophr. Res..

[12] Sadler I., Bauer A., Kassam S. (2024). Dietary habits and self-reported health outcomes in a cross-sectional survey of health-conscious adults eating a plant-based diet. J. Hum. Nutr. Diet..

[13] Wiśniewska K., Okręglicka K.M., Jaworski M., Nitsch-Osuch A. (2025). Plant-based vs. animal-based diets: Appetitive traits and dietary patterns in adults based on cross-sectional surveys. Nutrients.

[14] Hopwood C.J., Bleidorn W., Schwaba T., Chen S. (2020). Health, environmental, and animal rights motives for vegetarian eating. PLoS ONE.

[15] Holler S., Cramer H., Liebscher D., Jeitler M., Schumann D., Murthy V., Michalsen A., Kessler C.S. (2021). Differences between omnivores and vegetarians in personality profiles, values, and empathy: A systematic review. Front. Psychol..

[16] Jeżewska-Zychowicz M., Gawęcki J., Wądołowska L., Czarnocińska J., Galiński G., Kołłajtis-Dołowy A., Roszkowski W., Wawrzyniak A., Przybyłowicz K., Stasiewicz B., Gawęcki J. (2024). KomPAN^®^ Kwestionariusz do badania poglądów i zwyczajów żywieniowych dla młodzieży w wieku 16–18 lat i dorosłych, wersja 2.2—Kwestionariusz do samodzielnego wypełnienia przez respondenta. KomPAN^®^ Kwestionariusz do Badania Poglądów i Zwyczajów Żywieniowych Oraz Procedura Opracowania Danych.

[17] Wądołowska L., Stasiewicz B., Gawęcki J. (2024). The manual for developing nutritional data from the KomPAN^®^ questionnaire. KomPAN^®^ Dietary Habits and Nutrition Beliefs Questionnaire and the Manual for Developing Nutritional Data.

[18] Majkowicz M., de Walden-Gałuszko K., Majkowicz M. (2000). Praktyczna ocena efektywności opieki paliatywnej-wybrane techniki badawcze. Ocena Jakości Opieki Paliatywnej w Teorii i Praktyce.

[19] Zigmond A.S., Snaith R.P. (1983). The hospital anxiety and depression scale. Acta Psychiatr. Scand..

[20] Wakabayashi A., Baron-Cohen S., Wheelwright S., Goldenfeld N., Delaney J., Fine D., Smith R., Weil L. (2006). Development of short forms of the Empathy Quotient (EQ-Short) and the Systemizing Quotient (SQ-Short). Pers. Individ. Differ..

[21] Jankowiak-Siuda K., Kantor-Martynuska J., Siwy-Hudowska A., Śmieja M., Dobrołowicz-Konkol M., Zaraś-Wieczorek I., Siedler A. (2017). Psychometric properties of the Polish adaptation of short form of the Empathy Quotient (EQ-Short). Psychiatr. Pol..

[22] IPAQ Research Committee (2005). Guidelines for Data Processing and Analysis of the International Physical Activity Questionnaire (IPAQ)-Short and Long Forms.

[23] Casas-Albertos E., Rodríguez-Martín N.M., Alcalá-Santiago Á., Sarriá B., Obón-Santacana M., García-Villanova B., Ruiz-López M.D., Requena-Mullor M., Jimenez-Zabala A., Rodriguez-Barranco M. (2026). Omnivorous and plant-based dietary patterns: A comparative analysis using data-driven and index-based approaches. Eur. J. Nutr..

[24] Dobersek U., Wy G., Adkins J., Altmeyer S., Krout K., Lavie C.J., Archer E. (2021). Meat and mental health: A systematic review of meat abstention and depression, anxiety, and related phenomena. Crit. Rev. Food Sci. Nutr..

[25] Peremin I., Gerić M., Jakaša I., Gajski G. (2026). DNA Damage Across Dietary Patterns: A Comet Assay Study in Vegans and Omnivores. Foods.

[26] Schrauzer G.N. (2002). Lithium: Occurrence, dietary intakes, nutritional essentiality. J. Am. Coll. Nutr..

[27] Molero P., De Lorenzi F., Gędek A., Strater C., Popescu E., Ortuño F., Van Der Does W., Martínez-González M.A., Molendijk M.L. (2025). Diet quality and depression risk: A systematic review and meta-analysis of prospective studies. J. Affect. Disord..

