# Supplementary material for: Beyond Dietary Identity: Habitual Food Consumption, Lifestyle Characteristics, and Mental Health in Adults Following Plant-Based Diets
Source: Nutrients. 2026 Sep 14;18(18):2997. doi: 10.3390/nu18182997 (PMC13610570; doi:10.3390/nu18182997)
Supplement: Supplementary file 1 [file nutrients-18-02997-s001.zip › nutrients-4525381-supplementary.pdf]

Supplementary Materials

Beyond Dietary Identity: Habitual Food Consumption, Lifestyle Characteristics, and Mental Health in Adults Following Plant-Based Diets

Tamara Kuświk, Konrad Janowski and Agnieszka Białek

Supplementary Table S1. All 33 habitual food-consumption comparisons.

Food group	Omnivorous median [IQR], times/day	Plant-based median [IQR], times/day	Rank-biserial r	p value	FDR q
White bread	0.50 [0.14, 1.00]	0.14 [0.06, 0.50]	-0.165	0.083	0.138
Wholemeal bread	0.50 [0.06, 0.50]	0.50 [0.06, 0.50]	-0.057	0.544	0.665
White rice/pasta/fine groats	0.14 [0.06, 0.50]	0.14 [0.06, 0.50]	-0.031	0.737	0.811
Whole grains/coarse groats	0.50 [0.06, 0.50]	0.50 [0.14, 0.50]	0.004	0.963	0.963
Fast food	0.06 [0.06, 0.06]	0.06 [0.06, 0.06]	-0.174	0.027	0.048
Fried foods	0.14 [0.06, 0.50]	0.14 [0.06, 0.50]	-0.212	0.023	0.045
Butter	0.50 [0.06, 1.00]	0.06 [0.00, 0.14]	-0.479	<0.001	<0.001
Lard	0.00 [0.00, 0.06]	0.00 [0.00, 0.00]	-0.286	<0.001	<0.001
Oils/margarines/mixes	0.06 [0.00, 0.50]	0.14 [0.00, 0.50]	0.040	0.678	0.771
Milk	0.50 [0.14, 1.00]	0.06 [0.00, 0.50]	-0.433	<0.001	<0.001
Fermented dairy	0.50 [0.14, 0.50]	0.14 [0.00, 0.50]	-0.330	<0.001	0.001
Cottage cheese	0.14 [0.06, 0.50]	0.06 [0.00, 0.14]	-0.364	<0.001	<0.001
Yellow/processed cheese	0.14 [0.06, 0.50]	0.14 [0.00, 0.50]	-0.227	0.017	0.038
Processed meats	0.50 [0.06, 0.50]	0.00 [0.00, 0.00]	-0.881	<0.001	<0.001
Red meat	0.14 [0.06, 0.50]	0.00 [0.00, 0.00]	-0.877	<0.001	<0.001
White meat	0.50 [0.14, 0.50]	0.00 [0.00, 0.00]	-0.988	<0.001	<0.001
Fish	0.06 [0.06, 0.14]	0.00 [0.00, 0.06]	-0.648	<0.001	<0.001
Eggs	0.50 [0.14, 0.50]	0.06 [0.00, 0.50]	-0.432	<0.001	<0.001
Legumes	0.06 [0.06, 0.14]	0.50 [0.14, 0.50]	0.656	<0.001	<0.001
Potatoes	0.14 [0.06, 0.50]	0.14 [0.06, 0.50]	0.010	0.912	0.940

Fruit	0.50 [0.50, 1.00]	1.00 [0.50, 2.00]	0.119	0.204	0.280
Vegetables	1.00 [0.50, 2.00]	2.00 [1.00, 2.00]	0.412	<0.001	<0.001
Sweets	0.50 [0.14, 0.50]	0.14 [0.06, 0.50]	-0.212	0.025	0.046
Instant/ready soups	0.00 [0.00, 0.06]	0.00 [0.00, 0.00]	-0.100	0.164	0.235
Canned meat	0.00 [0.00, 0.00]	0.00 [0.00, 0.00]	-0.248	<0.001	<0.001
Canned/pickled vegetables	0.06 [0.06, 0.14]	0.14 [0.06, 0.50]	0.232	0.013	0.031
Fruit juice	0.06 [0.00, 0.14]	0.06 [0.00, 0.14]	-0.048	0.605	0.713
Vegetable/mixed juice	0.06 [0.00, 0.06]	0.06 [0.00, 0.06]	0.128	0.156	0.233
Sweetened hot drinks	0.06 [0.00, 1.00]	0.06 [0.00, 1.00]	-0.075	0.419	0.554
Sugar-sweetened soft drinks	0.06 [0.00, 0.06]	0.00 [0.00, 0.06]	-0.205	0.023	0.045
Energy drinks	0.00 [0.00, 0.00]	0.00 [0.00, 0.00]	0.010	0.889	0.940
Water	2.00 [1.00, 2.00]	2.00 [2.00, 2.00]	0.119	0.089	0.140
Alcohol	0.06 [0.00, 0.06]	0.06 [0.00, 0.06]	-0.067	0.462	0.586
Positive rank-biserial r indicates higher habitual frequency in plant-based participants.					

Habitual frequencies are expressed as times/day using the KomPAN frequency conversion. Group comparisons used Mann–Whitney U tests. FDR q values were obtained using the Benjamini–Hochberg procedure across all 33 food-group comparisons. IQR, interquartile range; FDR, false discovery rate.

Supplementary Table S2. Revised hierarchy of HADS models for self-identified dietary status.

**Outcome**	**Model**	**N**	**b**	**HC3 SE**	**95% CI**	**p value**	**R^2**	**Adjusted R^2**
HADS-A	Crude	158	0.45	0.71	-0.95 to 1.85	0.529	0.003	-0.003
HADS-A	Minimally adjusted	158	0.43	0.72	-0.98 to 1.84	0.549	0.005	-0.008
HADS-A	Sociodemographic sensitivity	158	0.46	0.74	-1.00 to 1.92	0.537	0.050	0.019
HADS-A	Lifestyle sensitivity	158	0.37	0.73	-1.06 to 1.80	0.616	0.035	0.003
HADS-A	Phenotype-expanded	158	0.45	0.74	-1.00 to 1.89	0.545	0.016	-0.016
HADS-D	Crude	158	-0.48	0.59	-1.65 to 0.68	0.416	0.004	-0.002

HADS-D	Minimally adjusted	158	-0.46	0.60	-1.64 to 0.73	0.451	0.010	-0.003
HADS-D	Sociodemographic sensitivity	158	-0.33	0.64	-1.58 to 0.92	0.602	0.033	0.001
HADS-D	Lifestyle sensitivity	158	-0.50	0.59	-1.67 to 0.66	0.395	0.073	0.043
HADS-D	Phenotype-expanded	158	-0.20	0.63	-1.43 to 1.04	0.755	0.047	0.016

The coefficient b compares self-identified plant-based (vegetarian + vegan) with omnivorous dietary status. Crude models include dietary status only. Minimally adjusted models include dietary status and sex and constitute the revised primary adjusted models. Sociodemographic sensitivity models additionally include education, employment, and place of residence. Lifestyle sensitivity models include sex, smoking status, and alcohol-consumption frequency. Phenotype-expanded models include sex, BMI, DQI, and SSIE. OLS models used HC3 robust standard errors. Non-significant estimates were not interpreted as evidence of equivalence or absence of association.

Supplementary Table S3. Sensitivity of HADS estimates to alternative dietary classifications.

Outcome	Dietary classification	Model	N	b	95% CI	p value
HADS-A	Self-identified plant-based	Crude	158	0.45	-0.95 to 1.85	0.529
HADS-A	Self-identified plant-based	Minimal	158	0.43	-0.98 to 1.84	0.549
HADS-A	Meat/fish abstention	Crude	158	-0.57	-2.11 to 0.96	0.463
HADS-A	Meat/fish abstention	Minimal	158	-0.57	-2.12 to 0.98	0.470
HADS-A	Identity–meat/fish concordant plant-based status	Crude	158	-0.50	-2.06 to 1.07	0.534
HADS-A	Identity–meat/fish concordant plant-based status	Minimal	158	-0.49	-2.07 to 1.09	0.543
HADS-D	Self-identified plant-based	Crude	158	-0.48	-1.65 to 0.68	0.416
HADS-D	Self-identified plant-based	Minimal	158	-0.46	-1.64 to 0.73	0.451
HADS-D	Meat/fish abstention	Crude	158	-0.22	-1.56 to 1.12	0.753
HADS-D	Meat/fish abstention	Minimal	158	-0.22	-1.55 to 1.11	0.747
HADS-D	Identity–meat/fish concordant plant-based status	Crude	158	-0.25	-1.62 to 1.11	0.716

HADS-D	Identity–meat/fish concordant plant-based status	Minimal	158	-0.26	-1.61 to 1.09	0.705

Self-identified plant-based status was the primary exposure. Meat/fish abstention was defined as zero reported habitual frequency of processed meat, red meat, white meat, and fish. Identity–meat/fish concordant plant-based status required both self-identification as vegetarian/vegan and meat/fish abstention. Eggs and dairy were not components of the behavioral definition. Minimal models were adjusted for sex. A four-category identity × behavior regression was not fitted because only one self-identified omnivore met the meat/fish-abstention criterion, yielding an unstable cell (n=1).

Supplementary Table S4. Comparison of participants with valid and excluded IPAQ records.

Characteristic	Valid IPAQ (n=120)	Excluded IPAQ (n=38)	Test	p value	Effect	Effect type
BMI	24.57 ± 4.47	24.17 ± 3.99	Welch t	0.601	0.092	Cohen d
HADS-A	8.28 ± 3.81	9.18 ± 4.22	Welch t	0.241	-0.232	Cohen d
HADS-D	4.97 ± 3.56	5.32 ± 3.26	Welch t	0.585	-0.098	Cohen d
SSIE	25.18 ± 7.48	23.11 ± 7.58	Welch t	0.146	0.276	Cohen d
DQI	13.89 ± 11.72	12.60 ± 11.77	Welch t	0.559	0.109	Cohen d
Alcohol frequency (times/day)	0.06 [0.00, 0.06]	0.06 [0.00, 0.12]	Mann–Whitney U	0.158		
Plant-based identity	40/120 (33.3%)	13/38 (34.2%)	Fisher exact	1.000	0.962	Odds ratio
Female sex	101/120 (84.2%)	32/38 (84.2%)	Fisher exact	1.000	0.997	Odds ratio
Higher education	85/120 (70.8%)	19/38 (50.0%)	Fisher exact	0.030	2.429	Odds ratio
Employed/working	110/120 (91.7%)	33/38 (86.8%)	Fisher exact	0.358	1.667	Odds ratio
Residence >500k city	78/120 (65.0%)	22/38 (57.9%)	Fisher exact	0.445	1.351	Odds ratio
Smoking status (3-level)	current 29; former 33; never 58	current 9; former 13; never 16	Chi-square	0.709	0.066	Cramér V

Continuous variables are presented as mean ± SD except alcohol frequency, which is median [IQR]. Categorical variables are shown as n/N (%), except smoking status, for which category counts are given. Higher education was the only characteristic showing a statistically significant valid-versus-excluded difference in this prespecified screen. IPAQ, International Physical Activity Questionnaire; SSIE, Polish short form of the Empathy Quotient (EQ-Short); DQI, Diet Quality Index.

Supplementary Table S5. Dietary identity and reported animal-food consumption used to assess identity–behavior concordance.

Self-identified diet	N	Meat/fish abstainer	Non-abstainer	Processed meat >0	Red meat >0	White meat >0	Fish >0	Eggs >0	Milk >0	Fermented dairy >0	Cottage cheese >0	Cheese >0
Omnivorous	105	1 (1.0%)	104 (99.0%)	95 (90.5%)	93 (88.6%)	104 (99.0%)	96 (91.4%)	103 (98.1%)	92 (87.6%)	94 (89.5%)	90 (85.7%)	98 (93.3%)
Vegetarian	34	18 (52.9%)	16 (47.1%)	0 (0.0%)	1 (2.9%)	1 (2.9%)	16 (47.1%)	32 (94.1%)	26 (76.5%)	30 (88.2%)	29 (85.3%)	32 (94.1%)

Vegan	19	18 (94.7%)	1 (5.3%)	1 (5.3%)	0 (0.0%)	0 (0.0%)	0 (0.0%)	1 (5.3%)	4 (21.1%)	4 (21.1%)	1 (5.3%)	1 (5.3%)

Values are n (%) within each self-identified dietary group. Meat/fish abstention was defined as zero reported habitual frequency of processed meat, red meat, white meat, and fish. Among the 53 self-identified plant-based participants, 36 (67.9%) met this criterion and 17 (32.1%) did not. Among vegetarians, 18/34 (52.9%) were abstinent and 16/34 (47.1%) were non-abstinent; 15 of the 16 non-abstinent vegetarians reported fish consumption, and one reported red meat, white meat, and fish. Among vegans, 18/19 (94.7%) were abstinent and one reported processed-meat consumption. The KomPAN FFQ assessed habitual intake over the preceding year; reported discordance therefore should not automatically be interpreted as current misclassification. Egg and dairy variables are shown descriptively and were not part of the meat/fish-abstention definition.

Supplementary Table S6. IPAQ observed-case and inverse-probability-weighted HADS sensitivity analyses.

Outcome	Model	N	b	HC3 SE	95% CI	p value
HADS-A	Observed-case	120	0.23	0.84	-1.41 to 1.87	0.782
HADS-A	IPW	120	0.39	0.84	-1.26 to 2.05	0.640
HADS-D	Observed-case	120	-0.47	0.71	-1.86 to 0.92	0.509
HADS-D	IPW	120	-0.33	0.71	-1.73 to 1.07	0.646

Observed-case and IPW models included self-identified dietary status, sex, and standardized log1p-transformed IPAQ total physical activity. Stabilized inverse-probability-of-observation weights were estimated from dietary identity, sex, higher education, employment, residence in a city >500,000 inhabitants, smoking status, and alcohol-consumption frequency.

Supplementary Table S6B. Diagnostics for stabilized inverse-probability-of-observation weights.

Diagnostic	Value
Marginal P(IPAQ valid)	0.759
Weight mean	1.006
Weight SD	0.253
Weight min	0.859
Weight 5th percentile	0.873
Weight median	0.929
Weight 95th percentile	1.248
Weight 99th percentile	1.765
Weight max	3.300
Effective sample size	112.920

The effective sample size after weighting was 112.9 compared with 120 observed valid IPAQ records. The weight distribution was examined before interpretation of the weighted sensitivity models; the maximum stabilized weight was 3.30.
